# Training GPs to improve their management of work-related problems: results of a cluster randomized controlled trial

**DOI:** 10.1080/13814788.2018.1517153

**Published:** 2018-11-05

**Authors:** Cornelis A. de Kock, Peter L. B. J. Lucassen, Hans Bor, J. André Knottnerus, Peter C. Buijs, Romy Steenbeek, Antoine L. M. Lagro-Janssen

**Affiliations:** a Department of Primary and Community Care, Gender & Women’s Health, Radboud University Medical Center, Radboud Institute for Health Sciences, Nijmegen, The Netherlands;; b Department of Primary and Community Care, Radboud University Medical Center, Radboud Institute for Health Sciences, Nijmegen, The Netherlands;; c Department of General Practice, Maastricht University, Maastricht, The Netherlands;; d Health and Care, TNO Work, Leiden, The Netherlands;; e Health, Technology, TNO Work, Leiden, The Netherlands

**Keywords:** Work-related problems, general practitioners, randomized controlled trial, return-to-work self-efficacy, educational training

## Abstract

**Background:** Paying attention to their patients’ work and recognizing work-related problems is challenging for many general practitioners (GPs).

**Objectives:** To assess the effect of training designed to improve the care for patients with work-related problems in general practice.

**Methods:** A cluster randomized controlled trial among 32 Dutch GPs. GPs in the intervention group received five-hour training. GPs in the control group were not trained. Included patients (age 18–63, working ≥12 h per week) completed baseline questionnaires and follow-up questionnaires planned after one year. Primary outcome at patient level was patients’ expectations about their ability to work, measured using the return-to-work self-efficacy scale (RTW-SE). Primary outcomes on GP level were their use of ICPC-code Z05 (‘work-related problem’) per 1000 working-age patients and percentage of the electronic medical files of working-age patients in which information about occupation had been recorded.

**Results:** A total of 640 patients completed the baseline questionnaire and 281 the follow-up questionnaire. We found no statistically significant differences in patients’ RTW-SE scores: intervention 4.6 (95%CI: 4.2–5.0); control 4.5 (95%CI: 4.1–4.9). Twenty-nine GPs provided data about the GP-level outcomes, which showed no statistically significant differences: use of ICPC code Z05 11.6 (95%CI: 4.7–18.6) versus 6.0 (95%CI: –1.2 to 13.2) per 1000 working-age patients; recording of occupation 28.8% (95%CI: 25.8–31.7) versus 28.6% (95%CI: 25.6–31.6).

**Conclusion:** Training GPs did not improve patients’ work-related self-efficacy or GPs’ registration of work-related problems and occupation.

KEY MESSAGES

- Training GPs did not improve patients’ expectations concerning their ability to work.
- Training GPs did not increase recording of occupation and use of ICPC-code Z05.
- Educational training for GPs based on GPs’ needs, including personalized feedback, might improve care for patients with work-related problems.

## Introduction

Work is important for a person’s health and wellbeing [1,2]. Work-related problems (WRPs) can be problems caused by work but also problems caused by not being able to work. Both are prevalent among patients who visit general practitioners (GPs) [3,4]. GPs, expected to deliver person-centred care, should pay attention to possible WRPs when working-age patients visit them but they seem reluctant to take up this task [5–8]. This reluctance has been explained as a result of their conflicting roles. In most European countries GPs have to certify sick leave. This role as gatekeeper for social security benefits can be experienced as conflicting with their obligation to act in their patient’s interest. However, in the Netherlands, where occupational physicians (OPs) certify sick leave, GPs do not proactively address WRPs either. They were even said to have a ‘blind spot’ for work [5].

GPs, trusted by their patients and working at the point of entry into the healthcare system, are considered to be ideally positioned to quickly recognize WRPs and prevent avoidable sickness absence [5]. Therefore, governments adapted legislation and instigated the development of guidelines promoting collaboration and motivating GPs to pay more attention to advising on work modification [9]. Unfortunately, none of the measures taken so far were convincingly successful in making GPs proactively address WRPs [5,10–13].

Many strategies primarily aimed to reduce sick leave but one can argue whether this should be the main focus. That many people with serious health problems continue working illustrates how vital work can be for health and wellbeing [14,15]. People differ significantly in their expectations concerning the ability to work when faced with health problems. These expectations predict actual behaviour [16,17]. Apart from discussing sick leave, GPs should discuss their patients’ expectations to improve the patients’ sickness behaviour.

Training GPs can positively influence their performance and result in beneficial effects for patients. This was demonstrated in studies on domestic violence, advice on smoking cessation, and management of lower respiratory tract infections [18–20]. We assumed that training GPs would likewise increase their confidence in discussing patients’ work and improve their advice in the presence of WRPs. We also hypothesized that this would positively influence their patients’ expectations about their ability to work. Therefore, we carried out a cluster randomized controlled trial (RCT) addressing the following research questions: does training of GPs (a) positively influence the patients’ expectations concerning the ability to work; and (b) increase recording by the GPs of the occupation of patients and work-relatedness of their health problems?

## Methods

### Study design

This study was a cluster RCT among Dutch GPs about the effect of training to improve GPs’ care for patients with WRPs. The minimum follow-up time was one year. Each participating practice (one or more GPs) was a cluster. Participation of patients involved the completion of two questionnaires. The study was registered in ‘The Netherlands Trial Registry’ (number NTR3475) and its protocol published in 2014 [21].

### Recruitment of participants

In 2011, we sent a letter to all 1400 GPs working in the south-eastern part of the Netherlands inviting them to participate. As only six GPs had responded positively, personally approaching GPs through our networks resulted in 26 more GPs willing to participate. All worked more than two days per week as a GP.

Patients were invited to participate by the receptionists if they visited the GP during the study period (17 February 2012 to 31 January 2013). The receptionists asked them to fill out a form checking the inclusion criteria. The criteria were: age 18–63 years; paid work for at least 12 h per week; ability to read and complete questionnaires.

### Randomization and allocation concealment of practices

Randomization took place at practice level to prevent contamination. The researcher was blinded and matched a random allocation sequence (block size 2) with a randomly ordered list of participating practices. If more than one GP in a practice participated, we informed them about the condition they were allocated to, after all had confirmed participation.

### Intervention

The first training session took place on 16 February 2012 and lasted five hours. It consisted of lectures, small group discussions, and role-play. It covered six topics: (1) the connection between work and health; (2) usual care for WRPs and ways to improve it; (3) legislation regarding absenteeism and collaboration with OP; (4) gender aspects of work and WRPs; (5) activating care for patients with work-related distress; (6) registration of occupation and WRPs in the electronic medical record (EMR). The programme was based on the results of a focus group study, in which we found that lack of knowledge and counselling skills were barriers for GPs to identify and address WRPs proactively [22]. The programme ended with instructions about the recruitment of patients for the trial. All participants received the forms and questionnaires needed to invite patients and to collect data. Two GPs who were unable to attend this group session received adapted individual training by the first author (KK) in their practice. Two booster sessions took place in April and May 2012 and were attended by 14 GPs. Participants could present cases to a consulting OP and discuss ways to optimize registration of occupation and use of ICPC-code Z05.

### Outcomes

*Primary patient-level outcome: work-related self-efficacy.* Self-efficacy is a measure of a person’s expectations about his or her ability to execute a course of action [16]. Patients’ expectations concerning their ability to work, their work-related self-efficacy, were shown to predict actual working or taking sick leave better than illness-related measures. These expectations can be measured using the return-to-work self-efficacy scale (RTW-SE). In this 11-item scale, each item gets a score between 1 and 6 and the final score is the mean of the items, higher numbers indicate a higher return-to-work self-efficacy. Originally developed to measure the RTW-SE of patients on sick leave because of mental health problems, the authors considered it also relevant for patients with physical problems, and for patients who resumed their work [17].

*Primary GP-level outcomes: recording of WRP and occupation. *We used GPs’ use of the ICPC-code ‘Z05’ (for ‘work-related problem’) and their recording of information about occupation in the EMR as primary GP-level outcomes.

*Secondary GP level outcome: GP work-awareness*. We developed the ‘GP work-awareness scale’ (GWAS), to assess patients’ perception of their GP’s attention for work and WRPs. It ranges from 0–4 and is calculated by adding the values of the following four items (1 if positive and 0 if neutral or negative): (1) ‘does your GP know your occupation?’ (2) ‘did your GP discuss the possibility that your health problem is work-related?’ (3) ‘did your GP discuss sick leave?’ (4) ‘did your GP help finding solutions for WRP?’

*Other variables*. GPs’ experience; the following patient-reported characteristics (supplementary Table): work-relatedness of health problem; educational level; sector of employment; type of contract; working hours/week; monthly (individual) income; experienced health; presence of chronic illness; sick leave; visits to GP; visits to OP. The outcomes were described in more detail in the study protocol [21]. Not mentioned in the study protocol were two statements we asked patients to respond to (1) ‘I think it is important that my GP should know my occupation’ and (2) ‘I want advice from my GP about taking sick leave.’

### Data collection

*Patient questionnaires.* We assessed the RTW-SE, the GWAS and the ‘other variables’ using questionnaires. The receptionists asked patients meeting the inclusion criteria and consenting to participate, to fill out the baseline questionnaire (paper or online). We used LimeSurvey (LimeSurvey GmbH, Hamburg, Germany) software to process the questionnaire data. Twelve months after completion of the baseline questionnaire, research assistants invited all patients of whom addresses were available to fill out an identical follow-up questionnaire (paper or online), also the patients who had not completed the baseline questionnaire.

### EMR data

We assessed the primary outcomes on GP level using data extracted from the EMR of the participating GPs during two equal length periods (98 weeks) before and after the intervention. We assessed the use of ICPC-code Z05 by dividing the number of patients for whom this code was used during the pre- and post-periods, by the total number of working-age patients on the GP’s list, expressing the result as number per 1000. We assessed registration of occupation by taking a stratified random sample of 40 patients (20 female and 20 male; 10 of each gender aged 18–45 years and the other 10 aged 46–63 years), who had visited the GP during the post-intervention period. It was positive if at least one of six details about the patient’s occupation had been recorded: (1) employer; (2) sector; (3) occupation; (4) level; (5) working hours; (6) type of contract. We calculated the percentage of the sampled patients for whom registration of occupation was positive.

### Sample size estimation

We assumed that we had to be able to demonstrate a moderate effect, defined as half a standard deviation (SD) of the RTW-SE, with a power of 80% (α = 0.05; and assuming an intracluster correlation coefficient (ICC) = 0.15). In an earlier study, the mean score of the RTW-SE was 4.24 and the SD 1.14 [17]. Allowing for 25% attrition, we needed two groups of at least 12 GPs, recruiting 40 patients each (480 patients per group) to be able to demonstrate a difference of at least 0.57 on the RTW-SE.

### Statistical methods

The significance threshold was set at 0.05. We used *t*-tests and chi-square tests to assess differences between patients of both study groups. We also compared patients who completed both questionnaires with patients who were lost to follow-up. We calculated the ICC for the RTW-SE at baseline. To assess the effect of the intervention on the RTW-SE at follow-up, we used a multilevel analysis of covariance (ANCOVA) to calculate estimated marginal means [23]. We corrected for the baseline RTW-SE and for variables which were correlated (significance level *P* ≤0.25) with the RTW-SE: patient’s gender; age; educational level; sector of employment; type of contract; monthly income; weekly working hours; experienced health; chronic illness; sick leave; visits to GP; GP’s gender, GP’s experience.

For each GP, we assessed the use of ICPC-code Z05 and recording of occupation during the pre- and post-intervention period, and calculated the aggregated mean GWAS-score at baseline and follow-up. To estimate the effect of the intervention we used ANCOVA. Post-intervention and follow-up scores were dependent variables, study condition independent variable and post-intervention and baseline scores were covariates [23]. Data was analysed using SPSS 22 (IBM Corporation, Armonk, NY, USA).

## Results

### Population characteristics

*General practitioners.* Thirty-two GPs working in 26 practices agreed to participate in our study (intervention (I): 7 female (F)/9 male (M); control (C): 8F/8M. The mean number of years since qualification as GP was 17.0 (F 13.7; M 21.1). Two GPs withdrew from the study after the baseline measurement but one of them later permitted us to extract GP-level data from the EMR. Three other GPs, who continued the study, did not give this permission but one provided us with self-extracted ICPC-code Z05 data. For five GPs, no GWAS data were available at baseline or at follow-up. Therefore, we could analyse data on the use of ICPC code Z05 (WRPs) for 29 GPs, on registration of occupation for 28 GPs, and GWAS-data for 27 GPs.

**Figure 1.**
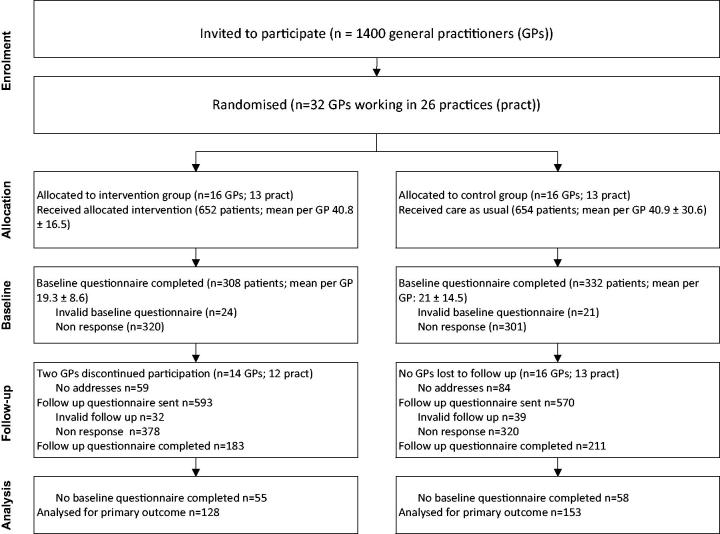
Flow chart of cluster RCT.

*Patients.* In total 1306 patients were enrolled; 640 completed the questionnaire at baseline; 281 at baseline and follow-up; 113 only at follow-up. Details are described in Figure 1. We found no significant differences between the study groups for the baseline characteristics (Table 1). One third (33.1%) indicated their health problem was possibly work-related and most agreed with the statements that GPs should know the occupation of patients (93.1%) and advise about sick leave (84.6%). The patients who were lost to follow-up were different in four respects: they were younger (43.3 vs 46.1; *P* <0.01); more were in the category low (15.4% vs 9.6%) or high education (34.5% vs 29.9%) (*P* = 0.02); more were self-employed (11.9% vs 6.5%) or temporarily employed (16.7% vs 11.8%) (*P* <0.01); had a monthly income between €1000 and €2000 (48.2% vs 40.6%) or did not report their income (11.9% vs 8.3%) whereas fewer earned between €2000 and €3000 (15.9% vs 25.5%) (*P* = 0.02).

**Table 1.** Baseline characteristics of all patients and of patients who completed both questionnaires.

	All patients			Patients who completed both questionnaires		
	Intervention	Control	*P*-value	Intervention	Control	*P*-value
*n*	308	332		128	153	
Female (%)	57.8	58.1	0.93	60.9	56.9	0.93
Age (mean ± SD)	43.9 ± 10.4	45.1 ± 11.1	0.15	45.4 ± 9.6	45.1 ± 11.1	0.32
Working hours per week (mean ± SD)	32.0 ± 10.5	31.2 ± 10.9	0.37	31.2 ± 11.0	30.6 ± 11.3	0.66
Could health problem be work-related? %						
* n*	307	328		128	151	
Yes	34.2	32.0	0.56	30.5	30.5	0.99
GP should know occupation of patients %						
* n*	308	332		128	153	
Yes	93.8	92.5	0.77	96.1	91.5	0.5
GP should advise taking sick leave %						
* n*	306	332		128	153	
Yes	85.6	83.7	0.77	86.7	81.7	0.52
Education						
* n*	307	331		128	153	
Low	12.4	13.3		8.6	10.5	
Middle	50.5	58.6	0.05	54.7	65.4	0.07
High	37.1	28.1		36.7	24.2	
Sector %						
* n*	301	326		127	150	
Blue collar	22.6	23.6		18.1	24.0	
White collar	43.5	41.7	0.9	40.9	38.0	0.49
Health; education; other professional^a^	33.9	34.7		40.9	38.0	
Employment %						
* n*	304	328		128	151	
Self-employed	9.5	9.4		5.5	7.3	
Permanent	75.1	75.8	0.92	82.8	80.8	0.82
Temporary	15.1	13.9		11.7	11.9	
Monthly income %						
* n*	303	328		127	151	
< €1000	16.5	20.1		16.5	21.2	
€1000–€2000	46.5	43.3		44.9	37.1	
€2000–€3000	21.1	19.2	0.73	27.6	23.8	0.32
> €3000	5.9	6.7		5.5	7.3	
Not known/no answer	9.9	10.7		5.5	10.6	
Experienced health %						
* n*	304	328		129	151	
Excellent	3.3	4.6		4.7	4.0	
Very good	17.8	14.9		15.5	13.2	
Good	59.2	62.5	0.62	61.2	65.6	0.9
Fair	18.4	17.4		17.1	16.6	
Poor	1.3	0.6		1.6	0.7	
Presence of chronic illness? %						
* n*	303	328		128	151	
Yes	39.6	39.9	0.93	37.5	43.7	0.29
Days of sick leave in the last 12 months %						
* n*	299	323		127	151	
0–5 days	68.6	70.3		66.9	69.5	
6–20 days	16.4	17.3	0.61	13.4	19.2	0.09
> 20 days	15.1	12.4		19.7	11.3	
Number of visits to general practitioner in the last six months %						
* n*	289	306		126	147	
None	7.3	10.8		5.6	9.5	
1 - 2	50.9	54.9	0.08	54.0	52.4	0.47
≥ 3	41.9	34.3		40.5	38.1	
Number of visits to occupational physician in the last six months %						
* n*	295	313		127	150	
None	80.7	85.0		78.7	84.0	
1	8.5	6.7	0.37	10.2	6.0	0.4
≥ 2	10.8	8.3		11.0	10.0	

a e.g. information technology professional, architect, copywriter etc. *P*-values were calculated using t-tests (for variables Age and Working hours per week) and chi square tests (all other variables).

### Primary patient level outcome: work-related self-efficacy

The mean RTW-SE was the same in both groups at baseline and at follow-up (4.7 vs 4.7; 4.9 vs 4.9). The ICC was 0.014. The estimated marginal means at follow-up was: intervention: 4.6 (95% CI: 4.2–5.0); control: 4.5 (95% CI: 4.1– 4.9) (Table 2).

**Table 2.** Baseline values and estimated marginal means at follow-up for primary patient-level outcome and primary and secondary GP-level outcomes.

	Intervention group		Control group		
	*n*		*n*		*P*-value
Primary patient-level outcome measureWork-related self-efficacy (RTW-SE)					
Value at baseline (mean ± SD)	308	4.7 ± 1.4	332	4.7 ± 1.2	0.84
Estimated marginal means at follow-up (95% CI)	128	4.6 (4.2–5.0)	153	4.5 (4.1–4.9)	0.74
Primary GP-level outcome measures					
Use of ICPC-code Z05 in EMR per 1000 working-age patients					
Value over pre-period (mean ± SD)	15	3.7 ± 4.4	14	4.8 ± 4.0	0.5
Estimated marginal means for post-period (95% CI)	15	11.6 (4.65 - 18.61)	14	6.0 (-1.22–13.23)	0.26
Percentage of patients with information about occupation in EMR					
Value over pre-period (mean ± SD)	14	13.9 ± 7.6	14	14.7 ± 7.4	0.8
Estimated marginal means for post-period (95% CI)	14	28.8 (25.83 - 31.74)	14	28.6 (25.64–31.55)	0.93
Secondary GP-level outcome measure					
GWAS (aggregated mean score)					
Value at baseline (mean ± SD)	16	2.2 ± 0.6	14	2.3 ± 0.6	0.77
Estimated marginal means at follow-up (95% CI)	14	2.5 (2.1–2.79)	13	2.1 (1.82 - 2.44)	0.1

SD, standard deviation; CI, confidence interval; EMR, electronic medical record; GWAS, GP work-awareness scale. *P*-values were calculated using t-test (for baseline values) and ANCOVA for (follow up and post-period values).

### Primary GP-level outcomes: recording of WRP and occupation

We found no statistically significant differences between the study groups (Table 2). Use of ICPC-code Z05 and registration of occupation were higher during the post-intervention period and the average registration of occupation doubled in both groups. The increase in the use of ICPC-code Z05 was larger in the intervention group (from 3.7 to 11.6/1000 vs 4.8 to 6.0/1000) but not statistically significant (*P* = 0.26).

### Secondary GP-level outcome: GP work-awareness

The difference between the GWAS score of GPs in both groups was not significant (I: 2.5; C: 2.1; *P* = 0.1).

## Discussion

### Main findings

In this cluster RCT, we found no statistically significant effect of training GPs on their patients’ work-related self-efficacy and on the recording of WRPs and occupation. About one-third of the participating patients assumed their problem was work-related. Almost all agreed with the statement that the GP should know their occupation and most wanted their GPs to advise sick leave.

### Comparison with the literature

Common factors in successful training programmes (recognition of intimate partner violence, smoking cessation, patient-centred consultation) were that they were targeted to the GPs’ individual needs and provided more opportunities to discuss barriers [18,19,24]. Probably, the lack of success in our trial can be partly attributed to our failure to target individual GPs’ needs concerning a proactive approach to patients with WRPs. Moreover, we did not provide feedback on their registration or GWAS-score. Finally, we did not sufficiently discuss the barriers and possible solutions [25–27]. That both groups increased their use of ICPC-code Z05 and even doubled their registration of occupation may be explained by a Hawthorne effect, as all participants knew the study was about work.

One-third of the patients indicated their health problem was possibly work-related. This prevalence is congruent with other studies. A recent French primary care based study found a prevalence of work-related common mental health problems among working-age patients of 26% and a systematic review reported similar numbers [3,15]. Insight into patients’ perception of their problem as work-related is essential for GPs, because there is much evidence for a positive relation between problems with returning to work and the cognition of the work-relatedness of the problem [28]. This is the more relevant in the light of our finding that Dutch patients want their GP to play an active role in WRPs.

Apart from individualizing the training and providing feedback, there is evidence that combining interventions may lead to better results. For example, a combination of peer group supervision, individualized telephone consultations, self-rating scales for professionals, and ongoing measurement of symptom severity in patients resulted in improved uptake of guidelines for the treatment of anxiety and depression by GPs [29].

### Strengths and limitations

To our knowledge, this is the first RCT studying the effect of training to improve GPs’ registration of WRPs and their care for patients with work-related problems. Recruiting working-age patients, regardless of the presence of WRPs, enabled us to estimate the work-related self-efficacy and prevalence of WRPs in the (Dutch) GPs’ waiting room population and to be informed about their opinion on GPs’ role in this field. A further strength was that we used outcome measures on both GP and patient levels, and that we assessed patients’ perception of GPs’ management of WRPs.

Several limitations may have contributed to the negative result of our trial. First, it was underpowered due to failure to include sufficient numbers of patients and loss to follow-up. Second, not limiting our study to patients experiencing WRPs possibly decreased the necessity for GPs to pay attention to work. This also resulted in a sample with a high baseline RTW-SE, limiting the room for improvement. Third, the outcome measures on a GP level may not reflect other changes in consultations occurring due to the training. Another limitation of the study is that the GWAS scale had not been validated.

### Implications for future research

Given the prevalence of WRPs among patients visiting their GP and the role patients see for GPs, we consider it relevant to study the care of GPs for patients with WRPs. In light of the demonstrated effectiveness of educational interventions in other fields, we recommend development of an intervention aimed at high-risk patients, tailored to the individual needs of the GP, and providing ongoing feedback.

## Conclusion

Training GPs did not increase patients’ work-related self-efficacy or GPs’ recording of WRPs or occupation; neither did patients experience more attention for work from GPs who were trained.
